# Grammar-aware phrase dataset generated using a novel python package

**DOI:** 10.1016/j.dib.2023.109237

**Published:** 2023-05-19

**Authors:** Ebisa A. Gemechu, G.R. Kanagachidambaresan

**Affiliations:** Department of Computer Science and Engineering, Vel Tech Rangarajan Dr. Sagunthala R&D Institute of Science and Technology, Chennai 600062, Tamil Nadu, India

**Keywords:** Oromo-grammar, Verb extraction, Machine translation, Grammar-aware, Oromo verb

## Abstract

The past technique of manual dataset preparation was time-consuming and needed much effort. Another attempt to the data acquisition method was using web scraping. Such web scraping tools also produce a bunch of data errors. For this reason, we developed “Oromo-grammar” a novel Python package that accepts a raw text file from the user, extracts every possible root verb from the text, and stores the verbs into a Python list. Our algorithm then iterates over list of root verbs to form their corresponding list of stems. Finally, our algorithm synthesizes grammatical phrases using the appropriate affixations and personal pronouns. The generated phrase dataset can indicate grammatical elements like numbers, gender, and cases. The output is a grammar-rich dataset, which is applicable to modern NLP applications like machine translation, sentence completion, and grammar and spell checker. The dataset also helps linguists and academia in teaching language grammar structures. The method can easily be reproducible to any other language with a systematic analysis and slight modifications to its affix structures in the algorithm.

**Specifications Table**

**Table:** Metadata Information about the prepared grammatical phrase dataset

Subject	Computer Science, Data Science
Specific subject area	Computer Science Applications, Data Engineering
Type of data	Text, Table
How the data were acquired	In this work, our objective is to design a system that automatically build a clean dataset for NLP in an efficient way. To do so, first, we collected a suitable Oromo raw text from online sources. We prepared the raw data into a single text file as an input. We designed a Python implemented software, that accepts the raw text, pre-process it, and generate the intended dataset. The system initially accepts the raw text as an input, extract all verbs from the text, trims the verbs into stems, and finally resynthesizes the stems with an appropriate affixes and personal pronouns. Eventually, the system generates a phrase-based grammatical dataset that is used for different NLP model training applications.
Data format	Raw and analyzed
Description of data collection	To prepare the dataset, we collected a suitable Oromo raw text from online sources. We used a sample of 200kb (about 100 pages) raw text to process the dataset. We prepared the data into a single text file and saved it into a local directory for data input. Before the data is extracted into tokens, we normalized the entire text using our pre-processing algorithm to remove white spaces, punctuations, and upper cases, which have no significant value to the intended data process.
Data source location	• Institution: Vel Tech Rangarajan Dr. Sagunthala R&D Institute of Science and Technology • City/Region: Chennai, Tamil Nadu • Country: India
Data accessibility	Repository name: Mendeley Data Data identification number: 10.17632/n5wg3mbp9r.1 Direct URL to data: https://data.mendeley.com/datasets/n5wg3mbp9r

## Value of the Data

• The dataset is used as the main input source to train neural networks and deep learning models in modern NLP applications like machine translation, sentence completion, and grammar and spell checker. It also helps linguists and academia in teaching language grammar structures. The dataset is not a mere collection of phrases. Rather, it's a grammar-aware dataset that can easily assist the machine to easily recognize syntactic elements or grammar rules during model training.
• This dataset benefits most researchers in NLP domain, especially to deal with low-resource languages. There are a few to none datasets available for low-resource languages for researchers to conduct research in NLP application. It gives the researchers the opportunity to directly dive into the research without the issue of getting data sources.
• The NLP researchers who focus on the Oromo language can directly use the dataset for sentence completion, and grammar and spell checker model training. They can also use it machine translation, after completing the English part of the phrases. The method can easily be reproducible to any other languages with a systematic analysis and slight modifications to the source code, specifically with the affixation structures of the other language to easily generate a similar dataset.

## 1 Objective

Over the past decades, machine translation (MT) and other NLP application has seen many advancements [1]. In the past, many researchers used Rule-based (RBMT) [2], Statistical Machine Translation (SMT) [3], and Neural Machine (NMT) methods [4]. Specifically, the last decade has seen many improvements using the NMT methods. However, those methods couldn't reach the quality of human translation [5]. Even the latest state-of-the-art NMT model is not as proficient as human translation. It's partly due to the lack of a suitable dataset to train the models. The situation is worse for under-resourced languages like Afaan Oromo [6].

The two commonly used datasets for language modes are rule-based and phrase-based. In practice, data preparation was used to be done manually by human translators, which needed much time and effort. The other method of data acquisition was using web scraping. This method also comes up with a bunch of data errors. Recent studies focused on seeking a solution to those errors [7,^8^,^9^,10]. Currently, users demand well-structured and summarized textual dataset [11]. Inspired by the unfulfilled data demand of NLP applications, specifically for low-resource languages, we build Oromo-grammar dataset [12]. The dataset is automatically generated using the custom Python package that we developed.

## 2 Data Description

So far, many researchers and the NLP community have heavily relied on manual dataset preparation for machine learning tasks. But this approach is tedious and time-consuming, since machine learning requires large dataset for the best performance. For most developed languages, datasets are readily available to be used by different NLP applications. However, for under-resourced languages, like Afaan Oromo, there were no publicly available datasets to build such applications. As already said, it's difficult to build a new dataset manually from the scratch. This is the main reason that motivated us to build this new dataset.

**Fig 1:**
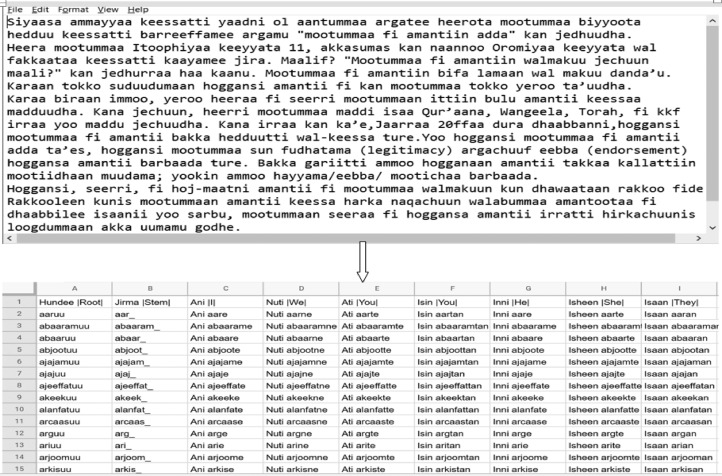
A screenshot of the raw text and the sample generated grammar-aware dataset.

Fig. 1 shows the sample input text consumed by the system, and the generated output as a csv file from the system. The screenshot portrayed here shows a very small snippet of the csv file that contains thousands of the system-generated dataset. When we give our system in kilobytes of text data, our system generates megabytes of a dataset from the given raw text.

As depicted by Fig. 1, the “root” column stores all the verb forms extracted from the raw text. The “stem” column stores all the list of trimmed stems from the coressponding verbs. The remaining columns show the derivatives of coressponding phrases generated by our system. Those phrase features will help as the useful source for different NLP applications.

## 3 Experimental Design, Materials and Methods

We developed a novel algorithm and methods to develop our software package using Python language. The algorithms are written in a logical flow to solve the problems: from text pre-processing, root word and stem formation, and dataset generation. The methods are shown by next Algorithms 1,2 and 3.

**Algorithm 1:** Text pre-processing.

***Input:****(in_filename), a raw text file saved as a .txt file in a directory* ***Output:****(filename), a cleaned text file saved as a .txt file in a directory.* ***steps:*** *1 import string* *2 def load_doc(filename):* *3 file ← open filename as a read-only* *4 text ← file.read()* *5 file.close()* *6 return text* *7 def clean_doc(doc):* *8 doc ← doc.replace('–', ' ')* *9 tokens← doc.split()* *10 table ← str.maketrans('', '', string.punctuation)* *11 tokens ← [w.translate(table) for w in tokens]* *12 tokens ← [word.lower() for word in tokens]* *13 return tokens* *14 def save_doc(lines, filename):* *15 data ← '\n'.join(lines)* *16 file ← open filename as read/write* *17 file.write(data)* *18 file.close()*

**Algorithm 2:** An algorithm that generates a grammar-aware Oromo phrase from the given raw text.

***Input:****(filename), a cleaned text file saved as a .txt file in a directory* ***Output:****(root_ve), unique list of root verb tokens* ***steps:*** *1 accept input from the user* *2 import os* *3 path ← " ", define and initialize the path* *4 def oro_text(path):* *5 in_filename ← input (complete file path from user input)* *7 print("Message to the user")* *8 return path* *9 user_text← oro_text(path)* *10 word_ext← clean_doc(user_text)* *11 oro_Vowels← ['a','e','i','o','u']* *12 verb_suffix← ['u','uu', 'uun','amu','e','ne','te','tan','an']* *13 OroStopWprds← [‘List of Stop Words]* *14 class Verb_extration:* *15 def root_verb(word_ext):* *16 next_verb ← [ ]* *17 for verb in word_ext:* *18 if verb not in OroStopWprds:* *19 [next_verb.append(verb) for suffix in verb_suffix if(suffix maches]* *20 return next_verb* *21 root_ve← Verb_extration.root_verb(word_ext)* *22 import numpy np* *23 root_ve ← list(np.unique(root_ve))* *24 print(root_ve[:10])*

**Algorithm 3:** Algorithm that creates a .csv file, generates data into a file, and downloads the csv file.

***Input:****(root_ve), The list of distinct root word tokens* ***Output:****(generated_grammar.csv), a csv file that contains a grammar-based of the generated dataset* ***steps:*** *1 import csv* *2 import files* *3 with open('generated_grammar.csv', 'w', newline='', encoding ='UTF-8′) as csvfile:* *4 fieldnames ← fieldnames (Insert Field names)* *5 writer ← csv.DictWriter(csvfile, fieldnames=fieldnames)* *6 writer.writeheader()* *7 for verb in root_ve:* *8 if((condition meets required affixes):* *9 continue* *10 Move over a series of ‘if-elif-else’ conditions to trim root verbs into their corresponding stems* *11 Concatenate the suitable affixes to the generated stems* *12 writer.writerow(‘Write the synthesized phrases into a csv file)* *13 files.download(generated_grammar.csv'), save the dataset file into ‘Downloads’*

The dataset is prepared using a custom Python algorithm. To prepare the dataset, we used a sample of 200KB (about 100 Pages of raw text) collected from online sources. Our algorithm performed well to automatically generate a grammar-aware dataset for the Oromo language. The method can easily be reproducible to any other language with a systematic analysis and slight modifications to its affix structures to generate similar datasets. The prepared dataset is a grammar-rich dataset, which is applicable to modern NLP applications like machine translation, sentence completion, and grammar and spell checker. The dataset also helps linguists and academia in teaching language grammar structures.

## Ethics Statements

Not applicable.

## CRediT Author Statement

**Ebisa A. Gemechu:** Conceptualization, Data curation, Formal analysis, Investigation, Methodology, Project administration, Resources, Software, Validation, Visualization, Writing – original draft, Writing; **G.R. Kanagachidambaresan:** Supervision, Validation, review & editing.

## Declaration of Competing Interest

The authors declare that they have no known competing financial interests or personal relationships that could have appeared to influence the work reported in this paper.

## Data Availability

Oromo Auto-Grammar Dataset (Original data) (Mendeley Data).
